# Weighted Estimation of the Accelerated Failure Time Model in the Presence of Dependent Censoring

**DOI:** 10.1371/journal.pone.0124381

**Published:** 2015-04-24

**Authors:** Youngjoo Cho, Debashis Ghosh

**Affiliations:** 1 Department of Statistics, The Pennsylvania State University, University Park, Pennsylvania, 16802, United States of America; 2 Department of Biostatistics and Informatics, Colorado School of Public Health, University of Colorado, Aurora, Colorado, 80045, United States of America; Brown University, UNITED STATES

## Abstract

Independent censoring is a crucial assumption in survival analysis. However, this is impractical in many medical studies, where the presence of dependent censoring leads to difficulty in analyzing covariate effects on disease outcomes. The semicompeting risks framework offers one approach to handling dependent censoring. There are two representative estimators based on an artificial censoring technique in this data structure. However, neither of these estimators is better than another with respect to efficiency (standard error). In this paper, we propose a new weighted estimator for the accelerated failure time (AFT) model under dependent censoring. One of the advantages in our approach is that these weights are optimal among all the linear combinations of the previously mentioned two estimators. To calculate these weights, a novel resampling-based scheme is employed. Attendant asymptotic statistical results for the estimator are established. In addition, simulation studies, as well as an application to real data, show the gains in efficiency for our estimator.

## Introduction

In medical studies, it is very common that death or withdrawal of study and progression on disease of interest simultaneously occur in the study. For this case, death or withdrawal of study may censor the development of disease. This type of data structure is called ‘semicompeting risks data’ [4].

Semicompeting risks data have been widely studied in the past decade. Some researchers used a Gamma copula to estimate the association parameter between the event of interest and dependent censoring [2], [4]. There is a literature that extended the methodology of [2] to the case that a nuisance parameter exists and also considered a more general copula model [22].

On the other hand, other researchers used semiparametric regression to model the event of interest and dependent censoring jointly. One approach is an estimation procedure based on the accelerated failure time (AFT) model [13], [17]. They used the artificial censoring technique to adjust the bias of the usual estimator. While the estimating equation of [13] is a U-statistic of order one, that of [17] is a U-statistic of order 2.

However, none of these papers fully discussed optimality of the estimator. In this case, choosing an estimator that is optimal from an efficiency viewpoint is an important issue for consideration. Here, we adapt the idea of [25], which proposed an optimal estimator whose form is a linear combination of estimators for multivariate failure time data. They used idea of [24], which proposed using combinations of dependent tests in the presence of missing values. Idea of [24] is to create a test which can maximize power based on linear combination of test statistics. Approach of [25] is simple and flexible, so it is sensible to apply their method in our case.

In this paper, we propose a weighted estimator by using methodology from [25]. Our weighted estimator combines those of [13] and [17]. The structure of this paper is as follows. In methods section, we review estimators proposed by [13] and [17] briefly. In addition, we describe details on our new weighted estimator. In model checking section, model checking procedure is briefly discussed. In simulation studies section, results of simulation studies will be given. Application of our method to a real data example is presented in real data analysis section. Some discussion concludes discussion section.

## Methods

### Review of Model

Let *X* be time to the event of interest, *D* the time to dependent censoring and *C* the time to independent censoring. All these times are transformed on a logarithmic scale. Let $\tilde{X}=X\wedge D\wedge C$ and $\tilde{D}=D\wedge C$. Define $\delta =I(X\leq \tilde{D})$, Δ = *I*(*D* ≤ *C*) and let **Z** be covariates. The data contain *n* independent and identically distributed observations $({\tilde{X}}_{i},{\tilde{D}}_{i},{\text{Z}}_{i},\delta_{i},\Delta_{i}),i=1,\ldots ,n$. The model is

$$\begin{array}{c} (\begin{array}{c} D_{i}=Z_{i}^{T}\eta_{0}+\epsilon_{i}^{D}\\ X_{i}=Z_{i}^{T}\theta_{0}+\epsilon_{i}^{X} \end{array}),\;i=1,\ldots ,n. \end{array}$$

where ***θ***_0_ and ***η***_0_ are *k* × 1 vectors, and $\epsilon_{i}\equiv (\epsilon_{i}^{X},\epsilon_{i}^{D})$ are error terms with an unknown joint distribution. In this case, we assume that the model is identifiable only in upper wedge *X* < *D* [4], [17]. We assume that *ϵ* has unknown distribution *H*. The goal is to obtain an unbiased estimator of ***α*** = (***η*** ^*T*^,***θ*** ^*T*^)^*T*^ without nonparametrically estimating the distribution of *ϵ*_*i*_, *i* = 1,…, *n*. We further assume that given **Z**, *C* and (*X*, *D*) are independent, but *X* and *D* can be dependent given **Z**. Now we are going to describe the procedures of [13] and [17] in turn.

Since $\tilde{D}$ only depends on independent censoring, a standard rank regression approach is available for estimation [11], [13], [15], [17], [20], [23]. The estimating equation for ***η*** is given by

$$\begin{array}{c} S_{n}\left(\eta \right)=n^{-1/2}\sum_{i=1}^{n}\Delta_{i}[Z_{i}-\frac{\sum_{j=1}^{n}Z_{j}I\left\{{\tilde{D}}_{j}^{*}\left(\eta \right)\geq {\tilde{D}}_{i}^{*}\left(\eta \right)\right\}}{\sum_{j=1}^{n}I\left\{{\tilde{D}}_{j}^{*}\left(\eta \right)\geq {\tilde{D}}_{i}^{*}\left(\eta \right)\right\}}], \end{array}$$

where ${\tilde{D}}_{i}^{*}(\eta )={\tilde{D}}_{i}-{\text{Z}}_{i}^{T}\eta$. The estimator of ***η*** can be obtained by solving **S**_*n*_(***η***) = 0.

For estimation of ***θ***, simply replacing ${\tilde{D}}_{i}-{\text{Z}}_{i}^{T}\eta$ to ${\tilde{X}}_{i}-{\text{Z}}_{i}^{T}\theta$ does not yield unbiased estimation of ***θ***. This is because the cause-specific hazard function for ${\tilde{X}}_{i}-{\text{Z}}_{i}^{T}\theta$ depends on ${\tilde{D}}_{i}-{\text{Z}}_{i}^{T}\theta$, which violates the model assumption [13]. To fix this problem, many authors use artificial censoring techniques [3], [6], [7], [10], [13], [17]. In [13], a single constant term *g*(***α***) is proposed so that the estimation equation will be unbiased for estimation of ***θ*** in the two-sample case. The form of *g*(***α***) is $g(\alpha )=\max_{1\leq i\leq n}\{0,{\text{Z}}_{i}^{T}(\theta -\eta )\}$. The proposed estimator in [13] is obtained by solving ${\text{U}}_{n}^{L}(\alpha )$ = 0, where

$$\begin{array}{ccc} U_{n}^{L}\left(\alpha \right) & = & n^{-1/2}\sum_{i=1}^{n}{\tilde{\delta }}_{i}^{*}\left(\alpha \right)[Z_{i}-\frac{\sum_{j=1}^{n}I\left\{{\tilde{X}}_{j}^{*}\left(\alpha \right)\geq {\tilde{X}}_{i}^{*}\left(\alpha \right)\right\}Z_{j}}{\sum_{j=1}^{n}I\left\{{\tilde{X}}_{j}^{*}\left(\alpha \right)\geq {\tilde{X}}_{i}^{*}\left(\alpha \right)\right\}}]\\ {\tilde{X}}_{i}^{*}\left(\alpha \right) & = & \left(X_{i}-Z_{i}^{T}\theta \right)\wedge \left(D_{i}-Z_{i}^{T}\eta -g\left(\alpha \right)\right)\wedge \left(C_{i}-Z_{i}^{T}\eta -g\left(\alpha \right)\right)\\ {\tilde{\delta }}_{i}^{*}\left(\alpha \right) & = & I\{\left(X_{i}-Z_{i}^{T}\theta \right)\leq \left(D_{i}-Z_{i}^{T}\eta -g\left(\alpha \right)\right)\wedge \left(C_{i}-Z_{i}^{T}\eta -g\left(\alpha \right)\right)\}, \end{array}$$ (1)

and *a*∧*b* means minimum of *a* and *b*. In [17], pairwise comparisons of all the subjects is proposed so that each subject has different degree of the artificial censoring. The transformations suggested by [17] are

$$\begin{array}{c} g_{ij}(\alpha )=\underset{{}_{i,j}}{\text{max}}\{0,Z_{i}^{T}(\theta -\eta ),Z_{j}^{T}(\theta -\eta )\}\\ {\tilde{X}}_{i(j)}^{*}(\alpha )=(X_{i}-Z_{i}^{T}\theta )\wedge (D_{i}-Z_{i}^{T}\eta -g_{ij}(\alpha ))\wedge (C_{i}-Z_{i}^{T}\eta -g_{ij}(\alpha ))\\ {\tilde{\delta }}_{i(j)}^{*}(\alpha )=I\{(X_{i}-Z_{i}^{T}\theta )\leq (D_{i}-Z_{i}^{T}\eta -g_{ij}(\alpha ))\wedge (C_{i}-Z_{i}^{T}\eta -g_{ij}(\alpha ))\}\\ \phi_{ij}(\alpha )={\tilde{\delta }}_{i(j)}^{*}(\alpha )I\{{\tilde{X}}_{i(j)}^{*}(\alpha )\leq {\tilde{X}}_{j(i)}^{*}(\alpha )\}-{\tilde{\delta }}_{j(i)}^{*}(\alpha )I\{{\tilde{X}}_{j(i)}^{*}(\alpha )\leq {\tilde{X}}_{i(j)}^{*}(\alpha )\}. \end{array}$$ (2)

The proposed estimator according to [17] is obtained by solving $U_{n}^{P}(\alpha )=0$, which is defined by

$$\begin{array}{c} U_{n}^{P}\left(\alpha \right)=\frac{2n^{1/2}}{n(n-1)}\sum_{1\leq i<j\leq n}\left(Z_{i}-Z_{j}\right)\Phi_{ij}\left(\alpha \right). \end{array}$$

Note that *X* and *D* are not observable, but we can express transformation (Eq 1) and (Eq 2) by using observable quantities [7], [13].

### Weighted estimator

Given these two estimation procedures, it is natural to consider their efficiencies with respect to standard error. However, in this point of view, neither estimator is superior to the other. Moreover, these estimators may not be optimal estimators with respect to the standard error. There is an argument that estimator of [17] gains more efficiency than that of [13] because pairwise comparisons lead to less artificial censoring than that in [13]. However, this logic only holds when we look at performance of estimators in the view of bias and variance across the estimators in simulation study. Concentrating on standard error of an estimator in a single dataset, the estimator by [17] may not provide better estimator than that of [13]. This will be seen in the real data analysis section.

The reason for this is due to estimation procedure of [17]. As discussed [7], for *n* samples, the number of comparisons of [13] for artificial censoring is of order *n*, while that of [17] is of order *n* ^2^. By definition of *g*_*ij*_(***α***), different degrees of artificial censoring is applied to observations. It may lead more variation between observations, which makes standard error larger than that of [13].

Having discussed our data structure and estimators from [13] and [17], we now describe the proposed estimation in this paper. Let $\hat{\eta }={\left({\hat{\eta }}_{1},\ldots ,{\hat{\eta }}_{k}\right)}^{T}$ be estimator of ***η***_0_, ${\hat{\theta }}^{L}={\left({\hat{\theta }}_{1}^{L},\ldots ,{\hat{\theta }}_{k}^{L}\right)}^{T}$ be estimator of ***θ***_0_ by [13] and ${\hat{\theta }}^{P}={\left({\hat{\theta }}_{1}^{P},\ldots ,{\hat{\theta }}_{k}^{P}\right)}^{T}$ be estimator of ***θ***_0_ by [17]. ${\hat{\theta }}^{L}$ and ${\hat{\theta }}^{P}$ are asymptotically unbiased estimators of ***θ***_0_.

We extend the scope of estimators which provide consistent estimation of ***θ***_0_. The natural extension of estimators of [13] and [17] is to consider collections of estimators that are linear combination of these two estimators with sum of weights being 1. By choosing proper weights, we can expect that the variance of the new combined estimator is smaller than that of each individual estimator in ${\hat{\theta }}^{L}$ and ${\hat{\theta }}^{P}$.

The goal is to find weights such that the variance of the new estimator is smaller than the minimum of variance of the estimators by [13] and [17], which have good theoretical properties. To obtain the estimator that yields smallest variance with these properties, we can use the idea of [25], which was applied to the problem of modeling multivariate failure times.

In [25], the joint distribution of estimators $\hat{\gamma }=\{{\hat{\gamma }}_{mr}\}$ is considered, where *m* = 1,…, *k* and *r* = 1…*R*. In this case, *m* indicates index of regression parameters and *r* stands for index of the *r*th event. For obtaining an optimal estimator, they applied arguments from [24] which derived a linear combination of test statistic to maximize power against every alternative hypothesis. Let $\hat{\text{H}}$ be the covariance matrix for the estimators $\hat{\gamma }$. Then we fix *m* and define ${\hat{\text{H}}}_{m}$ be covariance matrix of ${\hat{\gamma }}_{m}=({\hat{\gamma }}_{m1},\ldots ,{\hat{\gamma }}_{mR})$. It can be obtained from the entire covariance matrix by selecting the part corresponding to $\hat{\gamma }$ for *r* = 1,…, *R* under fixed *m*. Now we can define $\sum_{r=1}^{R}d_{r}{\hat{\gamma }}_{mr}$, where **d** = (*d*_1_, *d*_2_,…, *d*_*R*_) satisfies $\sum_{r=1}^{R}d_{r}=1$ [25]. Then $\text{d}\equiv {\left({\text{e}}^{T}{\hat{\text{H}}}_{m}^{-1}\text{e}\right)}^{-1}{\hat{\text{H}}}_{m}^{-1}\text{e}$ is a vector of weights which leads the best estimator among linear combinations of estimators of ${\hat{\gamma }}_{m}$ where **e** is a vector consisting of *R* ones [24], [25].

We now apply the argument in previous paragraph to our model by considering the joint distribution of $\hat{\beta }={\left\{{\hat{\eta }}^{T},{\left({\hat{\theta }}^{L}\right)}^{T},{\left({\hat{\theta }}^{P}\right)}^{T}\right\}}^{T}$. Let $\beta_{0}={\left(\eta_{0}^{T},\theta_{0}^{T},\theta_{0}^{T}\right)}^{T}$ and $G_{n}(\beta )={\left[S_{n}^{T}(\eta ),{\left\{U_{n}^{L}(\alpha )\right\}}^{T},{\left\{U_{n}^{P}(\alpha )\right\}}^{T}\right]}^{T}$ where ${\left[S_{n}^{T}(\eta ),{\left\{U_{n}^{L}(\alpha )\right\}}^{T},{\left\{U_{n}^{P}(\alpha )\right\}}^{T}\right]}^{T}$ are estimating equations for ***β***_0_. The strong consistency and asymptotic joint distribution of three estimators, described in following theorems, play a crucial role in our methodology.

To prove asymptotic results, several regularity conditions are required. As stated in [7] and [17], define

$$\begin{array}{c} F\left(a,b,c,d,e\right)=P\left(\epsilon_{1}^{X}-\epsilon_{1}^{D}\leq a,\epsilon_{1}^{X}-C_{1}\leq b,\epsilon_{1}^{X}-\epsilon_{2}^{X}\leq c,\epsilon_{1}^{X}-\epsilon_{2}^{D}\leq d,\epsilon_{1}^{X}-C_{2}\leq e|Z_{1},Z_{2}\right) \end{array}$$

Let $\alpha_{0}={\left(\eta_{0}^{T},\theta_{0}^{T}\right)}^{T}$. Define

$$\begin{array}{ccc} T_{1}\left(Z_{1},Z_{2}\right) & = & \frac{\partial F}{\partial a}\left\{g_{12}\left(\alpha_{0}\right),-Z_{1}^{T}\eta_{0}-g_{12}\left(\alpha_{0}\right),0,g_{12}\left(\alpha_{0}\right),-Z_{2}^{T}\eta_{0}-g_{12}\left(\alpha_{0}\right)\right\}\\ & & +\;\frac{\partial F}{\partial b}\left\{g_{12}\left(\alpha_{0}\right),-Z_{1}^{T}\eta_{0}-g_{12}\left(\alpha_{0}\right),0,g_{12}\left(\alpha_{0}\right),-Z_{2}^{T}\eta_{0}-g_{12}\left(\alpha_{0}\right)\right\}\\ & & +\;\frac{\partial F}{\partial d}\left\{g_{12}\left(\alpha_{0}\right),-Z_{1}^{T}\eta_{0}-g_{12}\left(\alpha_{0}\right),0,g_{12}\left(\alpha_{0}\right),-Z_{2}^{T}\eta_{0}-g_{12}\left(\alpha_{0}\right)\right\}\\ & & +\;\frac{\partial F}{\partial e}\left\{g_{12}\left(\alpha_{0}\right),-Z_{1}^{T}\eta_{0}-g_{12}\left(\alpha_{0}\right),0,g_{12}\left(\alpha_{0}\right),-Z_{2}^{T}\eta_{0}-g_{12}\left(\alpha_{0}\right)\right\}\\ & & +\;2\frac{\partial F}{\partial c}\left\{g_{12}\left(\alpha_{0}\right),-Z_{1}^{T}\eta_{0}-g_{12}\left(\alpha_{0}\right),0,g_{12}\left(\alpha_{0}\right),-Z_{2}^{T}\eta_{0}-g_{12}\left(\alpha_{0}\right)\right\} \end{array}$$

and

$$\begin{array}{c} T_{2}\left(Z_{1},Z_{2}\right)=T_{1}\left(Z_{1},Z_{2}\right)-2\frac{\partial F}{\partial c}\left\{g_{12}\left(\alpha_{0}\right),-Z_{1}^{T}\eta_{0}-g_{12}\left(\alpha_{0}\right),0,g_{12}\left(\alpha_{0}\right),-Z_{2}^{T}\eta_{0}-g_{12}\left(\alpha_{0}\right)\right\} \end{array}$$

From the Appendix in [17], the additional conditions are as follows:

1. The parameter space 𝓦 is compact, and the true parameter ***α***_0_ is an interior point of 𝓦.
2. ***θ***_0_ is the only solution of the estimating equation $E\{n^{-1/2}{\text{U}}_{n}^{P}(\eta_{0},\theta )\}=0$.
3. *E*(||**Z**||^2^) < ∞, where ||·|| is Euclidean norm and there exists positive constant *K* such that partial derivatives of *F* are bounded by *K* and there exists positive constant K* such that marginal probability distribution of *F* is bounded by K* almost surely.
4. *cov*[(**Z**_1_ − **Z**_2_){*T*_1_(**Z**_1_, **Z**_2_)}^1/2^] and *cov*[(**Z**_1_ − **Z**_2_){*T*_2_(**Z**_1_, **Z**_2_)}^1/2^] are positive definite.

In many parts of proofs, we adapt arguments from [13] and [17].

**Theorem 1**. By conditions of *C*1 − *C*3 in Appendix of [17] and conditions in [27], $\hat{\beta }$ is (strongly) consistent.

*Proof*. Let $\hat{\beta }={\left\{{\hat{\eta }}^{T},{\left({\hat{\theta }}^{L}\right)}^{T},{\left({\hat{\theta }}^{P}\right)}^{T}\right\}}^{T}$. It suffices to show that $\hat{\eta },{\hat{\theta }}^{L}$ and ${\hat{\theta }}^{P}$ are strongly consistent, respectively. Let ***α*** = (***η*** ^*T*^,***θ*** ^*T*^)^*T*^. Note that we have compact region, say 𝓦 and we assume regularity conditions in [27]. By [27], there exists nonrandom function *m*_1_ such that sup_***η*** ∈ 𝓝_0__||*n* ^−1/2^ **S**_*n*_(***η***) − **m**_1_(***η***)|| converges to 0 with probability 1 where 𝓝_0_ is a neighborhood of ***η***_0_. Thus $\hat{\eta }$ is strongly consistent. Similarly, we have another nonrandom function **m**_2_ such that $\sup_{\alpha \in {𝓝}_{1}}||n^{-1/2}{\text{U}}_{n}^{L}(\alpha )-{\text{m}}_{2}(\alpha )||$ converges to 0 with probability 1 where 𝓝_1_ is a neighborhood of ***α***_0_. Hence by [27], ${\hat{\alpha }}^{L}$ is strongly consistent.

For ${\hat{\theta }}^{P}$, by argument in Appendix of [17], note that by the U-statistics version of the law of large numbers, for all ***α*** ∈ 𝓦, $\left||n^{-1/2}{\text{U}}_{n}^{P}(\alpha )-\gamma (\alpha )|\right|$ converges to 0 in probability where $\gamma (\alpha )=E\{n^{-1/2}{\text{U}}_{n}^{P}(\alpha )\}$. We can partition our compact space as 𝓦_1_,…,𝓦_*k*_ so that $𝓦\in \cup_{j=1}^{k}{𝓦}_{j}$. Clearly, then for {***α*** ^*j*^ ∈ 𝓦_*j*_, *j* = 1,…, *k*}, $\max_{1\leq j\leq k}||n^{-1/2}{\text{U}}_{n}^{P}(\alpha^{j})-\gamma (\alpha^{j})||$ converges to 0 in probability. Then by Appendix of [17],

$$\begin{array}{ccc} & & \underset{||\alpha -\alpha^{**}\left|\right|\leq \xi }{\sup}n^{-1/2}\left|\right|U_{n}^{P}\left(\alpha \right)-U_{n}^{P}\left(\alpha^{**}\right)\left|\right|\\ & & \;\leq \frac{2}{n(n-1)}\sum_{1\leq i<j\leq n}\left|\right|Z_{i}-Z_{j}\left|\right|\underset{||\alpha -\alpha^{**}\left|\right|\leq \xi }{\sup}\left|\Phi_{ij}\left(\alpha \right)-\Phi_{ij}\left(\alpha^{**}\right)\right| \end{array}$$

and for all *ϵ* > 0, there exists *ξ* > 0 such that

$$\begin{array}{c} \lim_{n\to \infty }P(\frac{2}{n(n-1)}\sum_{1\leq i<j\leq n}\left|\right|Z_{i}-Z_{j}\left|\right|\underset{||\alpha -\alpha^{**}\left|\right|\leq \xi }{\sup}\left|\Phi_{ij}\left(\alpha \right)-\Phi_{ij}\left(\alpha^{**}\right)\right|\geq \epsilon )=0 \end{array}$$

Hence

$$\begin{array}{c} \lim_{n\to \infty }P(\underset{||\alpha -\alpha^{**}\left|\right|\leq \xi }{\sup}n^{-1/2}\left|\right|U_{n}^{P}\left(\alpha \right)-U_{n}^{P}\left(\alpha^{**}\right)||\geq \epsilon )=0 \end{array}$$

Thus ${\hat{\theta }}^{P}$ is strongly consistent and clearly, $\hat{\beta }$ is strongly consistent.

**Theorem 2**. Assuming certain technical conditions from [27] and [17], $n^{1/2}(\hat{\beta }-\beta_{0})$ is asymptotically normal with mean zero vector and covariance matrix **Σ**_0_ where $\Sigma_{0}=\Gamma_{0}^{-1}\Omega_{0}\Gamma_{0}^{-1},$ where **Γ**_0_ is a nonsingular matrix and **Ω**_0_ is the asymptotic covariance matrix of **G**_*n*_(***β***_0_).

*Proof*. As consistency, we assume the same regularity conditions as in [27]. Let $\beta_{0}={\left(\eta_{0}^{T},\theta_{0}^{T},\theta_{0}^{T}\right)}^{T}$ and $G_{n}(\beta )={\left[S_{n}^{T}(\eta ),{\left\{U_{n}^{L}(\alpha )\right\}}^{T},{\left\{U_{n}^{P}(\alpha )\right\}}^{T}\right]}^{T}$. Similar to [13], let $\lambda_{0}^{\left(1\right)}(t)$ be the cause-specific hazard function for the ${\tilde{D}}_{i}^{*}(\eta )$ and let $\lambda_{0}^{\left(2\right)}(t)$ be the cause-specific hazard function for ${\tilde{X}}_{i}^{*}(\alpha )$ under dependent censoring. Define

$$\begin{array}{c} M_{1i}\left(t\right)=N_{1i}\left(t;\eta_{0}\right)-\int_{-\infty }^{t}I\left\{{\tilde{D}}_{i}^{*}\left(\eta_{0}\right)\geq u\right\}\lambda_{0}^{\left(1\right)}\left(u\right)du \end{array}$$ (3)

$$\begin{array}{c} M_{2i}\left(t\right)=N_{2i}\left(t;\alpha_{0}\right)-\int_{-\infty }^{t}I\left\{{\tilde{X}}_{i}^{*}\left(\alpha_{0}\right)\geq u\right\}\lambda_{0}^{\left(2\right)}\left(u\right)du \end{array}$$ (4)

Then *M*_1*i*_ and *M*_2*i*_ are martingales [5], [13]. By adapting a proof in the Appendix in [13], Rebdolledo’s martingale central limit theorem [5] gives

$$\begin{array}{c} S_{n}\left(\eta_{0}\right)=n^{-1/2}\sum_{i=1}^{n}\int_{-\infty }^{\infty }\left\{Z_{i}-{\bar{Z}}^{\left(1\right)}\left(u\right)\right\}dM_{1i}\left(u\right)+o_{p}\left(1\right) \end{array}$$

$$\begin{array}{c} U_{n}^{L}\left(\alpha_{0}\right)=n^{-1/2}\sum_{i=1}^{n}\int_{-\infty }^{\infty }\left\{Z_{i}-{\bar{Z}}^{\left(2\right)}\left(u\right)\right\}dM_{2i}\left(u\right)+o_{p}\left(1\right) \end{array}$$

where ${\bar{\text{Z}}}^{\left(1\right)}(u)=\lim_{n\to \infty }[\sum_{j=1}^{n}I\{{\tilde{D}}_{j}^{*}(\eta_{0})\geq u\}{\text{Z}}_{j}]/[\sum_{j=1}^{n}I\{{\tilde{D}}_{j}^{*}(\eta_{0})\geq u\}]$ and ${\bar{\text{Z}}}^{\left(2\right)}(u)=\lim_{n\to \infty }[\sum_{j=1}^{n}I\{{\tilde{X}}_{j}^{*}(\alpha_{0})\geq u\}{\text{Z}}_{j}]/[\sum_{j=1}^{n}\{I({\tilde{X}}_{j}^{*}(\alpha_{0})\geq u)\}]$. From Appendix of [17],

$$\begin{array}{c} U_{n}^{P}\left(\alpha_{0}\right)=n^{-1/2}\sum_{i=1}^{n}2h_{1}\left(V_{i},\alpha_{0}\right)+o_{p}\left(1\right) \end{array}$$

where 2**h**_1_(**v**,***α***_0_) = 2*E*[**h**(**v**,**V**_2_,***α***_0_)]. For *j* = 1,…, *n*, *M*_1*j*_(*t*) is the martingale associated with $\epsilon_{j}^{D}$, while *M*_2*j*_(*t*) is the martingale associated with $\epsilon_{j}^{X}$ and **h**(**V**_*i*_,**V**_*j*_,***α***) = (**Z**_*i*_ − **Z**_*j*_)*ϕ*_*ij*_(***α***) [13], [17]. For *j* = 1,…, *n*, define

$$\begin{array}{c} a_{0j}=\int_{-\infty }^{\infty }\left\{Z_{j}-{\bar{Z}}^{\left(1\right)}\left(u\right)\right\}dM_{1j}\left(u\right)\;a_{1j}=\int_{-\infty }^{\infty }\left\{Z_{j}-{\bar{Z}}^{\left(2\right)}\left(u\right)\right\}dM_{2j}\left(u\right) \end{array}$$

$$\begin{array}{c} a_{2j}=2h_{1}\left(V_{j},\alpha_{0}\right), \end{array}$$

By the Cramér-Wold theorem, **G**_*n*_(***β***_0_) has an asymptotically normal distribution with mean zero and covariance matrix **Ω**_0_, where

$$\begin{array}{c} \Omega_{0}=E(\begin{array}{ccc} a_{01}a_{01}^{T} & a_{01}a_{11}^{T} & a_{01}a_{21}^{T}\\ a_{11}a_{01}^{T} & a_{11}a_{11}^{T} & a_{11}a_{21}^{T}\\ a_{21}a_{01}^{T} & a_{21}a_{11}^{T} & a_{21}a_{21}^{T} \end{array}) \end{array}$$

Note that $E\{n^{-1/2}{\text{U}}_{n}^{P}(\alpha )\}=\gamma (\alpha )$. As stated in the Appendix of [17], under conditions of *N*1 − *N*3 from [9], there exists an open neighborhood of ***α***_0_, say *K*_0_, such that

$$\begin{array}{c} \underset{\alpha \in K_{0}}{\sup}\frac{\left|\right|U_{n}^{P}\left(\alpha \right)-U_{n}^{P}\left(\alpha_{0}\right)-n^{1/2}\gamma \left(\alpha \right)\left|\right|}{1+n^{1/2}\left||\gamma \left(\alpha \right)|\right|}=o_{p}\left(1\right) \end{array}$$ (5)

Using a Taylor series expansion of **γ**(***α***) around ***α***_0_,

$$\begin{array}{c} \gamma \left(\alpha \right)=\gamma \left(\alpha_{0}\right)+\frac{\partial \gamma (\alpha )}{\partial \eta }{|}_{\alpha =\alpha_{0}}\left(\eta -\eta_{0}\right)+\frac{\partial \gamma (\alpha )}{\partial \theta }{|}_{\alpha =\alpha_{0}}\left(\theta -\theta_{0}\right)+o(||\alpha -\alpha_{0}\left||\right) \end{array}$$ (6)

With these two results (Eq 5) and (Eq 6), by Appendix of [17],

$$\begin{array}{c} U_{n}^{P}\left(\alpha \right)=U_{n}^{P}\left(\alpha_{0}\right)+n^{1/2}\frac{\partial \gamma (\alpha )}{\partial \eta }{|}_{\alpha =\alpha_{0}}\left(\eta -\eta_{0}\right)+n^{1/2}\frac{\partial \gamma (\alpha )}{\partial \theta }{|}_{\alpha =\alpha_{0}}\left(\theta -\theta_{0}\right)+o_{p}(1+n^{1/2}||\alpha -\alpha_{0}\left||\right) \end{array}$$ (7)

From [27], we have that

$$\begin{array}{c} S_{n}\left(\eta \right)=S_{n}\left(\eta_{0}\right)+n^{1/2}P_{0}\left(\eta -\eta_{0}\right)+o_{p}\left(1\right) \end{array}$$ (8)

for any ***η*** in the small neighborhood of ***η***_0_, where **P**_0_ is *k* × *k* nonsingular matrix. From the Appendix in [13], for $J_{1n}(\alpha )={\left[S_{n}^{T}(\eta ),{\left\{U_{n}^{L}(\alpha )\right\}}^{T}\right]}^{T}$,

$$\begin{array}{c} J_{1n}\left(\alpha \right)=J_{1n}\left(\alpha_{0}\right)+n^{1/2}L_{10}\left(\alpha -\alpha_{0}\right)+o_{p}\left(1\right) \end{array}$$ (9)

for any ***α*** in the small neighborhood of ***α***_0_, where **L**_10_ is defined as

$$\begin{array}{c} L_{10}=(\begin{array}{cc} P_{0} & 0\\ M_{0} & H_{0} \end{array}) \end{array}$$

is 2*k* × 2*k* nonsingular matrix and **M**_0_ and **H**_0_ are *k* × *k* constant matrices. Define $J_{2n}(\alpha )={\left[S_{n}^{T}(\eta ),{\left\{U_{n}^{P}(\alpha )\right\}}^{T}\right]}^{T}$. Using expansion from [17], for any ***α*** in the small neighborhood of ***α***_0_,

$$\begin{array}{c} J_{2n}\left(\alpha \right)=J_{2n}\left(\alpha_{0}\right)+n^{1/2}L_{20}\left(\alpha -\alpha_{0}\right)+o_{p}\left(1\right) \end{array}$$ (10)

$$\begin{array}{c} L_{20}=(\begin{array}{cc} P_{0} & 0\\ R_{0} & V_{0} \end{array}) \end{array}$$

where ${\text{R}}_{0}=\frac{\partial \gamma (\alpha )}{\partial \eta }{|}_{\alpha =\alpha_{0}}$ and ${\text{V}}_{0}=\frac{\partial \gamma (\alpha )}{\partial \theta }{|}_{\alpha =\alpha_{0}}$. Combining expansions of (Eq 8), (Eq 9) and (Eq 10), we have

$$\begin{array}{c} G_{n}\left(\beta \right)=G_{n}\left(\beta_{0}\right)+n^{1/2}\Gamma_{0}\left(\beta -\beta_{0}\right)+o_{p}\left(1\right) \end{array}$$

for any ***β*** in the small neighborhood of ***β***_0_, where **Γ**_0_ is defined as

$$\begin{array}{c} \Gamma_{0}=(\begin{array}{ccc} P_{0} & 0 & 0\\ M_{0} & H_{0} & 0\\ R_{0} & 0 & V_{0} \end{array}) \end{array}$$

The results from [9] and [27], along with the consistency of $\hat{\beta }$, imply that

$$\begin{array}{c} n^{1/2}\left(\hat{\beta }-\beta_{0}\right)=-\Gamma_{0}^{-1}G_{n}\left(\beta_{0}\right)+o_{p}\left(1\right) \end{array}$$

By combining the above results with Slutsky’s theorem, $n^{1/2}(\hat{\beta }-\beta_{0})$ has an asymptotically normal distribution with mean zero and covariance matrix $\Gamma_{0}^{-1}\Omega_{0}\Gamma_{0}^{-1}$.

Theorem 2 implies the asymptotic normality of $\hat{\beta }$ with the form of **Σ**_0_ being

$$\begin{array}{c} \Sigma_{0}=(\begin{array}{ccc} \Sigma_{11} & \Sigma_{12} & \Sigma_{13}\\ \Sigma_{21} & \Sigma_{22} & \Sigma_{23}\\ \Sigma_{31} & \Sigma_{32} & \Sigma_{33} \end{array}). \end{array}$$

Let $\hat{\Sigma }$ be the estimated covariance matrix of **Σ**_0_. In this covariance matrix, ${\hat{\Sigma }}_{11}$ is a *k* × *k* covariance matrix for $\hat{\eta }$, ${\hat{\Sigma }}_{22}$ is a *k* × *k* covariance matrix for ${\hat{\theta }}^{L}$ and ${\hat{\Sigma }}_{33}$ is a *k* × *k* covariance matrix for ${\hat{\theta }}^{P}$. Moreover, ${\hat{\Sigma }}_{12}$ and ${\hat{\Sigma }}_{13}$ represent covariance terms between $\hat{\eta }$ and ${\hat{\theta }}^{L}$ and between $\hat{\eta }$ and ${\hat{\theta }}^{P}$, respectively. Define ${\hat{\Sigma }}_{23}$ as the covariance matrix between ${\hat{\theta }}^{L}$ and ${\hat{\theta }}^{P}$. Clearly, ${\hat{\Sigma }}_{21}={\hat{\Sigma }}_{12}^{T}$, ${\hat{\Sigma }}_{31}={\hat{\Sigma }}_{13}^{T}$ and ${\hat{\Sigma }}_{32}={\hat{\Sigma }}_{23}^{T}$.

The issue remains of how to obtain the matrix corresponding to ${\hat{\text{H}}}_{m}^{-1}$ in our context. Note that $\hat{\eta },{\hat{\theta }}^{L}$ and ${\hat{\theta }}^{P}$ are correlated with each other. The estimating equation structure implies that ${\hat{\theta }}^{L}$ and ${\hat{\theta }}^{P}$ cannot be estimated separately from $\hat{\eta }$. Thus our matrix corresponding to ${\hat{\text{H}}}_{m}^{-1}$ should include the effect of $\hat{\eta }$. To obtain the matrix, we need to invert whole matrix and extract submatrix corresponding to ${\hat{\theta }}^{L}$ and ${\hat{\theta }}^{P}$. There are two approaches to obtain submatrix.

The first approach is to invert $\hat{\Sigma }$ and obtain the submatrix of ${\hat{\Sigma }}^{-1}$ corresponding to ${\hat{\theta }}_{m}^{L}$ and ${\hat{\theta }}_{m}^{P}$. Let us denote this matrix as ${\hat{\Sigma }}_{m}^{*}$. Clearly, this matrix is 2 × 2 and also positive definite. Then we can calculate ${\hat{\text{c}}}_{m}={\left({\hat{c}}_{m1},{\hat{c}}_{m2}\right)}^{T}={\left({\text{h}}^{T}{\hat{\Sigma }}_{m}^{*}\text{h}\right)}^{-1}{\hat{\Sigma }}_{m}^{*}\text{h}$, where **h** = (1, 1)^*T*^. By using the form of the optimal estimator in [25], we obtain new weighted estimator for *m*th covariate, say ${\hat{\theta }}_{m}^{MWE}$, where

$$\begin{array}{c} {\hat{\theta }}_{m}^{MWE}={\hat{c}}_{m1}{\hat{\theta }}_{m}^{L}+{\hat{c}}_{m2}{\hat{\theta }}_{m}^{P}. \end{array}$$

We can repeat this step for the other regression coefficients. Then we obtain ${\hat{\theta }}^{MWE}={\left({\hat{\theta }}_{1}^{MWE},\ldots ,{\hat{\theta }}_{k}^{MWE}\right)}^{T}$. In this first approach, weights are generated through using *k* number of 2 × 2 matrices. We can refer this first approach as ‘marginal approach’.

Sometimes it is desirable to consider entire covariates all at once when obtaining weights. The second approach is to obtain the corresponding submatrix of ${\hat{\Sigma }}^{-1}$ for ${\left\{{\left({\hat{\theta }}^{L}\right)}^{T},{\left({\hat{\theta }}^{P}\right)}^{T}\right\}}^{T}$. We denote this matrix as ${\hat{\Sigma }}^{**}$. This approach is different from first one in that ${\hat{\Gamma }}_{m}$ consists of elements of the covariance matrix from ${\hat{\theta }}_{m}^{L}$ and ${\hat{\theta }}_{m}^{P}$ but now ${\hat{\Sigma }}^{**}$ has elements of covariance matrix from corresponding entire ${\left\{{\left({\hat{\theta }}^{L}\right)}^{T},{\left({\hat{\theta }}^{P}\right)}^{T}\right\}}^{T}$. This approach reflects the effect of ${\left\{{\left({\hat{\theta }}^{L}\right)}^{T},{\left({\hat{\theta }}^{P}\right)}^{T}\right\}}^{T}$ jointly on our new estimator. Let **E** be a 2*k* × *k* matrix such that

$$\begin{array}{c} E=(\begin{array}{c} 1,0,\ldots ,0\\ 0,1,\ldots ,0\\ \vdots \\ 0,0,\ldots ,1\\ 1,0,\ldots ,0\\ 0,1,\ldots ,0\\ \vdots \\ 0,0,\ldots ,1 \end{array}). \end{array}$$

**E** is a multivariate extension of **h**. Note that **E** is concatenation of two *k* × *k* identity matrices by row. Entries that are 1 in these two *k* × *k* identity matrices are source of weights for ${\hat{\theta }}^{L}$ and ${\hat{\theta }}^{P}$. The next step is to construct $\hat{\text{B}}$, which is

$$\begin{array}{c} \hat{B}={\left\{{\left(E^{T}{\hat{\Sigma }}^{**}E\right)}^{-1}E^{T}{\hat{\Sigma }}^{**}\right\}}^{T} \end{array}$$

Then $\hat{\text{B}}$ has the form

$$\begin{array}{c} (\begin{array}{ccc} {\hat{c}}_{1,1}^{*} & \ldots & {\hat{c}}_{1,k}^{*}\\ {\hat{c}}_{2,1}^{*} & \ldots & {\hat{c}}_{2,k}^{*}\\ \vdots & \vdots & \vdots \\ {\hat{c}}_{(k+1),1}^{*} & \ldots & {\hat{c}}_{(k+1),k}^{*}\\ \vdots & \vdots & \vdots \\ {\hat{c}}_{2k,1}^{*} & \ldots & {\hat{c}}_{2k,k}^{*} \end{array}). \end{array}$$

This matrix is a multivariate extension of ${\hat{\text{c}}}_{m}$ from the first approach. This matrix is a contrast matrix in the sense that ${\hat{c}}_{m,m}^{*}+{\hat{c}}_{(k+m),m}^{*}=1$ for the *m*th regression coefficient of ${\hat{\theta }}^{L}$ and ${\hat{\theta }}^{P}$. Moreover, ${\hat{c}}_{p,p}^{*}+{\hat{c}}_{(k+p),p}^{*}=0$ for *p* ≠ *m* = 1,…, *k*. Using a vector form, from this approach our new estimator, say ${\hat{\theta }}^{JWE}$,

$$\begin{array}{c} {\hat{\theta }}^{JWE}={\left({\hat{\theta }}_{1}^{JWE},\ldots ,{\hat{\theta }}_{k}^{JWE}\right)}^{T}={\left({\hat{c}}_{1,1}^{*}{\hat{\theta }}_{1}^{L}+{\hat{c}}_{(k+1),1}^{*}{\hat{\theta }}_{1}^{P},\ldots ,{\hat{c}}_{k,k}^{*}{\hat{\theta }}_{k}^{L}+{\hat{c}}_{(2k),k}^{*}{\hat{\theta }}_{k}^{P}\right)}^{T}. \end{array}$$

We can also refer this approach as the ‘joint approach’.

Now the key step is to obtain $\hat{\Sigma }$. We use the resampling approach of [16], which was also used in [13] and [17]. Let ${\hat{\alpha }}^{L}={\left\{{\hat{\eta }}^{T},{\left({\hat{\theta }}^{L}\right)}^{T}\right\}}^{T}$ and ${\hat{\alpha }}^{P}={\left\{{\hat{\eta }}^{T},{\left({\hat{\theta }}^{P}\right)}^{T}\right\}}^{T}$. From [13] and [17], we have

$$\begin{array}{ccccc} W_{i}^{\left(1\right)} & = & \Delta_{i}[Z_{i}-\frac{\sum_{j=1}^{n}I\left\{{\tilde{D}}_{j}^{*}\left(\hat{\eta }\right)\geq {\tilde{D}}_{i}^{*}\left(\hat{\eta }\right)\right\}Z_{j}}{\sum_{j=1}^{n}I\left\{{\tilde{D}}_{j}^{*}\left(\hat{\eta }\right)\geq {\tilde{D}}_{i}^{*}\left(\hat{\eta }\right)\right\}}] & & -\;\sum_{l=1}^{n}\frac{\Delta_{l}I\left\{{\tilde{D}}_{i}^{*}\left(\hat{\eta }\right)\geq {\tilde{D}}_{l}^{*}\left(\hat{\eta }\right)\right\}}{\sum_{j=1}^{n}I\left\{{\tilde{D}}_{j}^{*}\left(\hat{\eta }\right)\geq {\tilde{D}}_{l}^{*}\left(\hat{\eta }\right)\right\}}\\ & & \times \;[Z_{i}-\frac{\sum_{j=1}^{n}I\left\{{\tilde{D}}_{j}^{*}\left(\hat{\eta }\right)\geq {\tilde{D}}_{l}^{*}\left(\hat{\eta }\right)\right\}Z_{j}}{\sum_{j=1}^{n}I\left\{{\tilde{D}}_{j}^{*}\left(\hat{\eta }\right)\geq {\tilde{D}}_{l}^{*}\left(\hat{\eta }\right)\right\}}], \end{array}$$

$$\begin{array}{ccc} W_{i}^{\left(2\right)} & = & {\tilde{\delta }}_{i}^{*}\left({\hat{\alpha }}^{L}\right)[Z_{i}-\frac{\sum_{j=1}^{n}I\left\{{\tilde{X}}_{j}^{*}\left({\hat{\alpha }}^{L}\right)\geq {\tilde{X}}_{i}^{*}\left({\hat{\alpha }}^{L}\right)\right\}Z_{j}}{\sum_{j=1}^{n}I\left\{{\tilde{X}}_{j}^{*}\left({\hat{\alpha }}^{L}\right)\geq {\tilde{X}}_{i}^{*}\left({\hat{\alpha }}^{L}\right)\right\}}]-\sum_{l=1}^{n}\frac{{\tilde{\delta }}_{l}^{*}\left({\hat{\alpha }}^{L}\right)I\left\{{\tilde{X}}_{i}^{*}\left({\hat{\alpha }}^{L}\right)\geq {\tilde{X}}_{l}^{*}\left({\hat{\alpha }}^{L}\right)\right\}}{\sum_{j=1}^{n}I\left\{{\tilde{X}}_{j}^{*}\left({\hat{\alpha }}^{L}\right)\geq {\tilde{X}}_{l}^{*}\left({\hat{\alpha }}^{L}\right)\right\}}\\ & & \times \;[Z_{i}-\frac{\sum_{j=1}^{n}I\left\{{\tilde{X}}_{j}^{*}\left({\hat{\alpha }}^{L}\right)\geq {\tilde{X}}_{l}^{*}\left({\hat{\alpha }}^{L}\right)\right\}Z_{j}}{\sum_{j=1}^{n}I\left\{{\tilde{X}}_{j}^{*}\left({\hat{\alpha }}^{L}\right)\geq {\tilde{X}}_{l}^{*}\left({\hat{\alpha }}^{L}\right)\right\}}], \end{array}$$

and

$$\begin{array}{c} W_{i}^{\left(3\right)}=\frac{2}{n-1}\sum_{j=1}^{n}\left(Z_{i}-Z_{j}\right)\Phi_{ij}\left({\hat{\alpha }}^{P}\right). \end{array}$$

Define

$$\begin{array}{c} W_{i}=(\begin{array}{c} W_{i}^{\left(1\right)}\\ W_{i}^{\left(2\right)}\\ W_{i}^{\left(3\right)} \end{array}). \end{array}$$

A consistent estimator of **Ω**_0_ is

$$\begin{array}{c} \hat{\Omega }=\frac{1}{n}\sum_{i=1}^{n}W_{i}W_{i}^{T}. \end{array}$$

We then solve the estimating equation

$$\begin{array}{c} G_{n}\left(\beta \right)=-n^{-1/2}\sum_{i=1}^{n}W_{i}Q_{i}, \end{array}$$ (11)

where *Q*_*i*_ (*i* = 1,…, *n*) represent standard normal random variables. Note that $G_{n}(\beta )={\left[S_{n}^{T}(\eta ),{\left\{U_{n}^{L}(\alpha )\right\}}^{T},{\left\{U_{n}^{P}(\alpha )\right\}}^{T}\right]}^{T}$ is joint estimating equation for ${\left(\eta_{0}^{T},\theta_{0}^{T},\theta_{0}^{T}\right)}^{T}$. By solving this equation, we obtain many realizations of $\hat{\beta }$s, say ${\hat{\beta }}^{R}={\left\{{\left({\hat{\eta }}^{*}\right)}^{T},{\left({\hat{\theta }}^{L*}\right)}^{T},{\left({\hat{\theta }}^{P*}\right)}^{T}\right\}}^{T}$ where ${\left\{{\left({\hat{\eta }}^{*}\right)}^{T},{\left({\hat{\theta }}^{L*}\right)}^{T},{\left({\hat{\theta }}^{P*}\right)}^{T}\right\}}^{T}$ are solutions from (Eq 11). The next theorem, combined with Theorem 2, justifies the resampling approach for calculating $\hat{\Sigma }$.

**Theorem 3**. Based on the technical conditions in [16], the unconditional distribution of $n^{1/2}(\hat{\beta }-\beta_{0})$ is same asymptotically as the conditional distribution of $n^{1/2}({\hat{\beta }}^{R}-\hat{\beta })$ where ${\hat{\beta }}^{R}$ are realizations of $\hat{\beta }$ from resampling.

*Proof*. Recall that for any ***β*** in the small neighborhood of ***β***_0_, we have

$$\begin{array}{c} G_{n}\left(\beta \right)=G_{n}\left(\beta_{0}\right)+n^{1/2}\Gamma_{0}\left(\beta -\beta_{0}\right)+o_{p}\left(1\right) \end{array}$$ (12)

Note that ${\hat{\beta }}^{R}$ are solutions of Eq (11). By conditioning on observed data and using expansion (Eq 12) as well as by adapting arguments in [13] and [16],

$$\begin{array}{c} G_{n}\left({\hat{\beta }}^{R}\right)=G_{n}\left(\hat{\beta }\right)+n^{1/2}\Gamma_{0}\left({\hat{\beta }}^{R}-\hat{\beta }\right)+o_{p}\left(1\right) \end{array}$$

and hence,

$$\begin{array}{c} n^{1/2}\left({\hat{\beta }}^{R}-\hat{\beta }\right)=-\Gamma_{0}^{-1}n^{-1/2}\sum_{i=1}^{n}W_{i}Q_{i}+o_{p}\left(1\right) \end{array}$$

Note that $n^{-1/2}\sum_{i=1}^{n}{\text{W}}_{i}Q_{i}$ is asymptotically normal with covariance matrix **Σ**_0_. Then given observed data, distribution of $n^{1/2}({\hat{\beta }}^{R}-\hat{\beta })$ is asymptotically normal with covariance matrix $\Gamma_{0}^{-1}\Sigma_{0}\Gamma_{0}^{-1}$. Hence conditional distribution of $n^{1/2}({\hat{\beta }}^{R}-\hat{\beta })$ on observed data is asymptotically same as unconditional distribution of $n^{1/2}(\hat{\beta }-\beta_{0})$.

For *m* = 1,…*k* and *j* = 1,…, *M*, let ${\left({\hat{\eta }}_{m}^{*}\right)}^{\left(j\right)},{\left({\hat{\theta }}_{m}^{L*}\right)}^{\left(j\right)}$ and ${\left({\hat{\theta }}_{m}^{P*}\right)}^{\left(j\right)}$ be *j*th realizations of an element ${\hat{\eta }}_{m},{\hat{\theta }}_{m}^{L}$ and ${\hat{\theta }}_{m}^{P}$ corresponding to *m*th covariate, respectively. The algorithm for the first approach is as follows.

1. By resampling, calculate the covariance matrix $\hat{\Sigma }$ using realizations ${\left({\hat{\eta }}_{m}^{*}\right)}^{\left(j\right)},{\left({\hat{\theta }}_{m}^{L*}\right)}^{\left(j\right)}$ and ${\left({\hat{\theta }}_{m}^{P*}\right)}^{\left(j\right)}$, (*m* = 1,…, *k* and *j* = 1,…, *M*).
2. From ${\hat{\Sigma }}^{-1}$, obtain the covariance matrix corresponding to ${\hat{\theta }}_{m}^{L}$ and ${\hat{\theta }}_{m}^{P}$, say ${\hat{\Sigma }}_{m}^{*}$.
3. Calculate ${\hat{\text{c}}}_{m}={\left({\hat{c}}_{m1},{\hat{c}}_{m2}\right)}^{T}={\left({\text{h}}^{T}{\hat{\Sigma }}_{m}^{*}\text{h}\right)}^{-1}{\hat{\Sigma }}_{m}^{*}\text{h}$ where **h** = (1,1)^*T*^ and obtain the new estimate ${\hat{\theta }}_{m}^{MWE}={\hat{c}}_{m1}{\hat{\theta }}_{m}^{L}+{\hat{c}}_{m2}{\hat{\theta }}_{m}^{P}$.
4. Repeat step 3 for all covariates.

The algorithm for the second approach is as follows.

1. By resampling, calculate the covariance matrix $\hat{\Sigma }$ using realizations ${\left({\hat{\eta }}_{m}^{*}\right)}^{\left(j\right)},{\left({\hat{\theta }}_{m}^{L*}\right)}^{\left(j\right)}$ and ${\left({\hat{\theta }}_{m}^{P*}\right)}^{\left(j\right)}$ (*m* = 1,…, *k* and *j* = 1,…, *M*).
2. Obtain ${\hat{\Sigma }}^{**}$ from $\hat{\Sigma }$.
3. From ${\hat{\Sigma }}^{**}$ and **E**, obtain $\hat{\text{B}}$.
4. Calculate the new estimate ${\hat{\theta }}_{m}^{JWE}={\hat{c}}_{m,m}^{*}{\hat{\theta }}_{m}^{L}+{\hat{c}}_{k+m,m}^{*}{\hat{\theta }}_{m}^{P}$, where ${\hat{c}}_{j,l}^{*}$ be the element of *j*th row and *l*th column of $\hat{\text{B}}$.

By Theorem 1 and Theorem 2, our new estimators are consistent and asymptotically normal.

## Model checking

For assessing the adequacy of the model, since our weight estimator is based on estimators from [13] and [17], it is reasonable to consider entire processes from [13] and [17]. In this case, we extend model checking technique from [13]. As defined in [13], Let $N_{1i}(t;\eta )=\Delta_{i}I\{{\tilde{D}}_{i}^{*}(\eta )\leq t\}$ and $N_{2i}(t;\alpha )={\tilde{\delta }}_{i}^{*}(\alpha )I\{{\tilde{X}}_{i}^{*}(\alpha )\leq t\}$, where *i* = 1,…, *n*. Then Nelson-Aalen estimators for the event of interest and dependent censoring are

$$\begin{array}{c} {\hat{\Lambda }}_{0}^{\left(1\right)}\left(u;\eta \right)=\int_{-\infty }^{t}\frac{\sum_{i=1}^{n}dN_{1i}\left(u;\eta \right)}{\sum_{j=1}^{n}I\left\{{\tilde{D}}_{j}^{*}\left(\eta \right)\geq u\right\}}\;{\hat{\Lambda }}_{0}^{\left(2\right)}\left(u;\alpha \right)=\int_{-\infty }^{t}\frac{\sum_{i=1}^{n}dN_{2i}\left(u;\alpha \right)}{\sum_{j=1}^{n}I\left\{{\tilde{X}}_{j}^{*}\left(\alpha \right)\geq u\right\}}. \end{array}$$

Note that by (Eq 3) and (Eq 4), martingale residuals are defined as

$$\begin{array}{c} {\hat{M}}_{1i}\left(t;\hat{\eta }\right)=N_{1i}\left(t;\hat{\eta }\right)-\int_{-\infty }^{t}I\left\{{\tilde{D}}_{i}^{*}\left(\hat{\eta }\right)\geq u\right\}d{\hat{\Lambda }}_{0}^{\left(1\right)}\left(u,\hat{\eta }\right) \end{array}$$

$$\begin{array}{c} {\hat{M}}_{2i}\left(t;\hat{\alpha }\right)=N_{2i}\left(t;\hat{\alpha }\right)-\int_{-\infty }^{t}I\left\{{\tilde{X}}_{i}^{*}\left(\hat{\alpha }\right)\geq u\right\}d{\hat{\Lambda }}_{0}^{\left(2\right)}\left(u,\hat{\alpha }\right), \end{array}$$

where $\hat{\alpha }$ can be either ${\hat{\alpha }}^{L}={\left\{{\hat{\eta }}^{T},{\left({\hat{\theta }}^{L}\right)}^{T}\right\}}^{T}$ or ${\hat{\alpha }}^{P}={\left\{{\hat{\eta }}^{T},{\left({\hat{\theta }}^{P}\right)}^{T}\right\}}^{T}$. Then as defined in [13],

$$\begin{array}{c} S_{n}\left(s;\eta \right)=n^{-1/2}\sum_{i=1}^{n}Z_{i}{\hat{M}}_{1i}\left(s;\eta \right)\;U_{n}\left(t;\alpha \right)=n^{-1/2}\sum_{i=1}^{n}Z_{i}{\hat{M}}_{2i}\left(t;\alpha \right). \end{array}$$

Then similar to [13] and [17], we can substitute $\hat{\eta }$ on **S**_*n*_(*s*; ***η***), ${\hat{\alpha }}^{L}$ and ${\hat{\alpha }}^{P}$ on **U**_*n*_(*t*;***α***). ${\left[S_{n}^{T}(s;\hat{\eta }),{\left\{U_{n}(t;{\hat{\alpha }}^{L})\right\}}^{T},{\left\{U_{n}(t;{\hat{\alpha }}^{P})\right\}}^{T}\right]}^{T}$ are called observed score processes with respect to dependent censoring and the event of interest, respectively [7], [13], [17]. We can construct ${\left[{\hat{S}}_{n}^{T}(s;{\hat{\eta }}^{*}),{\left\{{\hat{U}}_{n}^{L}(t;{\hat{\alpha }}^{L*})\right\}}^{T},{\left\{{\hat{U}}_{n}^{P}(v;{\hat{\alpha }}^{P*})\right\}}^{T}\right]}^{T}$ [13], [17], where

$$\begin{array}{ccc} {\hat{S}}_{n}\left(s;{\hat{\eta }}^{*}\right) & = & n^{-1/2}\sum_{i=1}^{n}\int_{-\infty }^{s}[Z_{i}-\frac{\sum_{j=1}^{n}I\left\{{\tilde{D}}_{j}^{*}\left(\hat{\eta }\right)\geq w\right\}Z_{j}}{\sum_{j=1}^{n}I\left\{{\tilde{D}}_{j}^{*}\left(\hat{\eta }\right)\geq w\right\}}]d{\hat{M}}_{1i}\left(w;\hat{\eta }\right)Q_{i}\\ & & +\;S_{n}\left(s;{\hat{\eta }}^{*}\right)-S_{n}\left(s;\hat{\eta }\right) \end{array}$$

$$\begin{array}{ccc} {\hat{U}}_{n}^{L}\left(t;{\hat{\alpha }}^{L*}\right) & = & n^{-1/2}\sum_{i=1}^{n}\int_{-\infty }^{t}[Z_{i}-\frac{\sum_{j=1}^{n}I\left\{{\tilde{X}}_{j}^{*}\left({\hat{\alpha }}^{L}\right)\geq w\right\}Z_{j}}{\sum_{j=1}^{n}I\left\{{\tilde{X}}_{j}^{*}\left({\hat{\alpha }}^{L}\right)\geq w\right\}}]d{\hat{M}}_{2i}\left(w;{\hat{\alpha }}^{L}\right)Q_{i}\\ & & +\;U_{n}\left(t;{\hat{\alpha }}^{L*}\right)-U_{n}\left(t;{\hat{\alpha }}^{L}\right) \end{array}$$

$$\begin{array}{ccc} {\hat{U}}_{n}^{P}\left(v;{\hat{\alpha }}^{P*}\right) & = & n^{-1/2}\sum_{i=1}^{n}\int_{-\infty }^{v}[Z_{i}-\frac{\sum_{j=1}^{n}I\left\{{\tilde{X}}_{j}^{*}\left({\hat{\alpha }}^{P}\right)\geq w\right\}Z_{j}}{\sum_{j=1}^{n}I\left\{{\tilde{X}}_{j}^{*}\left({\hat{\alpha }}^{P}\right)\geq w\right\}}]d{\hat{M}}_{2i}\left(w;{\hat{\alpha }}^{P}\right)Q_{i}\\ & & +\;U_{n}\left(v;{\hat{\alpha }}^{P*}\right)-U_{n}\left(v;{\hat{\alpha }}^{P}\right), \end{array}$$

where ${\hat{\alpha }}^{L*}={\left\{{\left({\hat{\eta }}^{*}\right)}^{T},{\left({\hat{\theta }}^{L*}\right)}^{T}\right\}}^{T}$ and ${\hat{\alpha }}^{P*}={\left\{{\left({\hat{\eta }}^{*}\right)}^{T},{\left({\hat{\theta }}^{P*}\right)}^{T}\right\}}^{T}$. These three processes are called bootstrapped processes [7], [13], [17]. We can plot the observed process with bootstrapped processes by randomly selecting 20 or 30 observations. Standard tests for goodness of fit can be performed by calculating Kolmogorov-Smirnov type test statistics. Test statistics are then defined by $\sup_{s}||{\text{S}}_{n}(s;\hat{\eta })||,\sup_{t}||{\text{U}}_{n}(t;{\hat{\alpha }}^{L})||$, and $\sup_{v}||{\text{U}}_{n}(v;{\hat{\alpha }}^{P})||$. To calculate the null distribution of the test statistics, first we obtain *j*th realizations of bootstrap samples ${\left({\hat{\eta }}^{*}\right)}^{\left(j\right)},{\left({\hat{\theta }}^{L*}\right)}^{\left(j\right)}$ and ${\left({\hat{\theta }}^{P*}\right)}^{\left(j\right)}$. Then we compute $BS_{j}=\sup_{s}||{\hat{\text{S}}}_{n}(s;{\left({\hat{\eta }}^{*}\right)}^{\left(j\right)})||,BS_{j}^{L}=\sup_{t}||{\hat{\text{U}}}_{n}^{L}(t;{\left({\hat{\alpha }}^{L*}\right)}^{\left(j\right)})||$ and $BS_{j}^{P}=\sup_{v}||{\hat{\text{U}}}_{n}^{P}(v;{\left({\hat{\alpha }}^{P*}\right)}^{\left(j\right)})||$, respectively for *j* = 1,…, *M*, where ${\left({\hat{\alpha }}^{L*}\right)}^{\left(j\right)}$ and ${\left({\hat{\alpha }}^{P*}\right)}^{\left(j\right)}$ are *j*th realizations of bootstrap samples of ${\hat{\alpha }}^{L*}$ and ${\hat{\alpha }}^{P*}$. The p-values can be defined by

$$\begin{array}{c} p_{1}=\frac{1}{M}\sum_{j=1}^{M}I\{BS_{j}\geq \underset{s}{\sup}\left|\right|S_{n}\left(s;\hat{\eta }\right)\left||\right\}\\ p_{2}=\frac{1}{M}\sum_{j=1}^{M}I\{BS_{j}^{L}\geq \underset{t}{\sup}\left|\right|U_{n}\left(t;{\hat{\alpha }}^{L}\right)\left||\right\}\\ p_{3}=\frac{1}{M}\sum_{j=1}^{M}I\{BS_{j}^{P}\geq \underset{v}{\sup}\left|\right|U_{n}\left(v;{\hat{\alpha }}^{P}\right)||\}. \end{array}$$

[10]. If the p-value is smaller than predetermined level, we reject the null hypothesis, which means that data does not have appropriate fit on our bivariate model. Note that a multiple testing problem arises for testing the models for ***θ***. We address this by adjusting p-values based on a Bonferroni correction with two tests.

## Simulation Studies

We consider two simulation settings. In first simulation setting, the errors follow a bivariate normal distribution with mean (0,1.2) with variance 1 and correlation *ρ* = 0,0.25. The independent censoring time *C* is generated from *log*(U*), where U* has uniform distribution with minimum value 0 and maximum value 20. Covariate is *Z* ∼ *Bernoulli*(0.5), where *Bernoulli*(0.5) is Bernoulli distribution with success probability 0.5. We run 500 simulation runs. Within each simulation run, 500 resampling runs are tried for covariance matrix calculation. Sample sizes are *N* = 150 and *N* = 300. If there is only one covariate in the model, the first and the second method of the weighted estimation are equivalent. Let this common weighed estimator be ${\hat{\theta }}^{WE}$. We calculate bias (Bias), mean squared error (MSE), mean of standard error (SEE), 95% coverage rate (Coverage). The coverage is based on the normal approximation. Moreover, to evaluate robustness of estimators, we also compute median of difference of the estimator from true value (Dmedian), median of squared error of estimates (Mediansq), and median of standard errors (Sdmedian). Results are summarized on Table 1 and Table 2.

**Table 1:** Simulation result when N = 150 and N = 300, ρ = 0 with covariate Bernoulli(0.5).

Estimators^1^	*N* = 150			
	Bias (Dmedian^2^)	MSE^3^ (Mediansq^4^)	SEE^5^(Sdmedian^6^)	Coverage^7^
${\hat{\theta }}^{L}$	0.018 (0.018)	0.04 (0.017)	0.204 (0.2)	0.95
${\hat{\theta }}^{P}$	0.021 (0.014)	0.036 (0.017)	0.193 (0.19)	0.96
${\hat{\theta }}^{WE}$	0.016 (0.006)	0.036 (0.015)	0.188 (0.185)	0.95
Estimators^1^	*N* = 300			
	Bias (Dmedian^2^)	MSE^3^(Mediansq^4^)	SEE^5^(Sdmedian^6^)	Coverage^7^
${\hat{\theta }}^{L}$	-0.002 (-0.003)	0.017 (0.006)	0.140 (0.140)	0.95
${\hat{\theta }}^{P}$	-0.001 (0.002)	0.016 (0.007)	0.133 (0.132)	0.95
${\hat{\theta }}^{WE}$	-0.004 (-0.002)	0.016 (0.007)	0.130 (0.129)	0.94

^1^ ${\hat{\theta }}^{L}$: the estimator by [13]; ${\hat{\theta }}^{P}$: the estimator by [17]; ${\hat{\theta }}^{WE}$: the weighted estimator by the proposed approach (Note that the marginal approach and the joint approach are equal in one variable case)

^2^ median of difference of the estimator from true value

^3^ mean squared error

^4^ median of squared error

^5^ mean of standard error

^6^ median of standard error

^7^ 95% coverage rate

**Table 2:** Simulation result when N = 150 and N = 300, ρ = 0.25 with covariate Bernoulli(0.5).

Estimators^1^	*N* = 150			
	Bias (Dmedian^2^)	MSE^3^(Mediansq^4^)	SEE^5^(Sdmedian^6^)	Coverage^7^
${\hat{\theta }}^{L}$	0.005 (0.01)	0.036 (0.017)	0.198 (0.197)	0.95
${\hat{\theta }}^{P}$	0.006 (0.007)	0.032 (0.015)	0.189 (0.188)	0.95
${\hat{\theta }}^{WE}$	-0.001 (-0.006)	0.033 (0.016)	0.184 (0.183)	0.94
Estimators	*N* = 300			
	Bias (Dmedian^2^)	MSE^3^(Mediansq^4^)	SEE^5^(Sdmedian^6^)	Coverage^7^
${\hat{\theta }}^{L}$	-0.003 (0.005)	0.018 (0.008)	0.138 (0.137)	0.95
${\hat{\theta }}^{P}$	0.001 (0.007)	0.017 (0.007)	0.131 (0.131)	0.95
${\hat{\theta }}^{WE}$	-0.003 (0.002)	0.017 (0.007)	0.129 (0.128)	0.95

^1^ ${\hat{\theta }}^{L}$: the estimator by [13]; ${\hat{\theta }}^{P}$: the estimator by [17]; ${\hat{\theta }}^{WE}$: the weighted estimator by the proposed approach (Note that the marginal approach and the joint approach are equal in one variable case)

^2^ median of difference of the estimator from true value

^3^ mean squared error

^4^ median of squared error

^5^ mean of standard error

^6^ median of standard error

^7^ 95% coverage rate

In second simulation setting, we generate Gamma random variable *ν* with mean *μ* = 1 and variance *σ* ^2^ = 0 or 1, then create *W* = exp (*ϵ* ^*X*^), which is an exponential random variable with rate 4*ν* ^−1^ and exp (*ϵ* ^*D*^) with an exponential random variable with rate *ν* ^−1^. Then we generate time to the event of interest by $\exp\left(X\right)=\exp\left(\theta_{0}^{T}\text{Z}\right)\exp\left(\epsilon^{X}\right)$ and time to the dependent censoring by $\exp\left(D\right)=\exp\left(\eta_{0}^{T}\text{Z}\right)\exp\left(\epsilon^{D}\right)$ (By notation in our paper, *X*, *D* and *C* are already log-transformed times. Thus in this context, exp (*X*), exp (*D*) and exp (*C*) are times in the original scale). The independent censoring time exp (*C*) has uniform distribution with minimum value 0 and maximum value 20. True parameter values are ***θ***_0_ = (0.5,1)^*T*^ and ***η***_0_ = (1,0.5)^*T*^ and covariates are *Z*_1_ ∼ *U*(0,1), where *U*(0,1) is uniform distribution with minimum value 0 and maximum value 1 and *Z*_2_ ∼ *Bernoulli*(0.5). We run 500 simulation runs. Within each simulation run, 500 resampling runs are tried for covariance matrix calculation. Let ${\hat{\theta }}^{MWE}$ be weighted estimators from calculating weights marginally (the first proposed method) and let ${\hat{\theta }}^{JWE}$ be weighted estimators from calculating weights jointly (the second proposed method). We compute the same quantities as we did in the first set of the simulation study. Results are summarized on Table 3 and Table 4.

**Table 3:** Simulation result when N = 150 and N = 300, σ^2^ = 0 with two covariates (Z_1_: U(0, 1), Z_2_: Bernoulli(0.5)).

Estimators^1^	*N* = 150							
	Bias (Dmedian^2^)		MSE^3^(Mediansq^4^)		SEE^5^(Sdmedian^6^)		Coverage^7^	
	*Z*_1_	*Z*_2_	*Z*_1_	*Z*_2_	*Z*_1_	*Z*_2_	*Z*_1_	*Z*_2_
${\hat{\theta }}^{L}$	0.0001 (0.002)	0.002 (-0.005)	0.12 (0.052)	0.042 (0.018)	0.358 (0.352)	0.226 (0.222)	0.96	0.96
${\hat{\theta }}^{P}$	-0.003 (0.003)	-0.003 (-0.002)	0.158 (0.074)	0.051 (0.023)	0.427 (0.418)	0.243 (0.241)	0.96	0.96
${\hat{\theta }}^{MWE}$	0.003 (-0.007)	0.003 (0.003)	0.123 (0.053)	0.043 (0.019)	0.351 (0.349)	0.219 (0.218)	0.95	0.95
${\hat{\theta }}^{JWE}$	0.003 (-0.007)	0.004 (0.001)	0.123 (0.055)	0.043 (0.018)	0.351 (0.349)	0.219 (0.217)	0.94	0.95
Estimators^1^	*N* = 300							
	Bias (Dmedian^2^)		MSE^3^(Mediansq^4^)		SEE^5^(Sdmedian^6^)		Coverage^7^	
	*Z*_1_	*Z*_2_	*Z*_1_	*Z*_2_	*Z*_1_	*Z*_2_	*Z*_1_	*Z*_2_
${\hat{\theta }}^{L}$	-0.012 (-0.013)	0.004 (0.001)	0.065 (0.028)	0.02 (0.01)	0.257 (0.255)	0.148 (0.148)	0.95	0.96
${\hat{\theta }}^{P}$	-0.014 (-0.017)	-0.001 (-0.015)	0.081 (0.035)	0.024 (0.013)	0.283 (0.281)	0.162 (0.162)	0.95	0.96
${\hat{\theta }}^{MWE}$	-0.01 (-0.012)	0.003 (-0.001)	0.064 (0.031)	0.02 (0.01)	0.252 (0.251)	0.146 (0.146)	0.95	0.96
${\hat{\theta }}^{JWE}$	-0.01 (-0.014)	0.003 (0.002)	0.064 (0.032)	0.02 (0.01)	0.251 (0.25)	0.146 (0.146)	0.95	0.96

^1^ ${\hat{\theta }}^{L}$: the estimator by [13]; ${\hat{\theta }}^{P}$: the estimator by [17]; ${\hat{\theta }}^{MWE}$: the weighted estimator by the marginal approach; ${\hat{\theta }}^{JWE}$: the weighted estimator by the joint approach

^2^ median of difference of the estimator from true value

^3^ mean squared error

^4^ median of squared error

^5^ mean of standard error

^6^ median of standard error

^7^ 95% coverage rate

**Table 4:** Simulation result when N = 150 and N = 300, σ^2^ = 1 with two covariates (Z_1_: U(0,1), Z_2_: Bernoulli(0.5)).

Estimators^1^	*N* = 150							
	Bias (Dmedian^2^)		MSE^3^(Mediansq^4^)		SEE^5^(Sdmedian^6^)		Coverage^7^	
	*Z*_1_	*Z*_2_	*Z*_1_	*Z*_2_	*Z*_1_	*Z*_2_	*Z*_1_	*Z*_2_
${\hat{\theta }}^{L}$	-0.010 (-0.003)	-0.033 (-0.039)	0.273 (0.127)	0.086 (0.038)	0.441 (0.434)	0.315 (0.312)	0.90	0.96
${\hat{\theta }}^{P}$	0.002 (-0.002)	-0.032 (-0.046)	0.295 (0.142)	0.095 (0.038)	0.559 (0.552)	0.325 (0.324)	0.95	0.96
${\hat{\theta }}^{MWE}$	-0.009 (-0.008)	-0.030 (-0.031)	0.263 (0.128)	0.085 (0.041)	0.437 (0.432)	0.303 (0.301)	0.90	0.96
${\hat{\theta }}^{JWE}$	-0.009 (-0.008)	-0.030 (-0.03)	0.262 (0.128)	0.086 (0.04)	0.437 (0.432)	0.303 (0.301)	0.90	0.96
Estimators^1^	*N* = 300							
	Bias (Dmedian^2^)		MSE^3^(Mediansq^4^)		SEE^5^(Sdmedian^6^)		Coverage^7^	
	*Z*_1_	*Z*_2_	*Z*_1_	*Z*_2_	*Z*_1_	*Z*_2_	*Z*_1_	*Z*_2_
${\hat{\theta }}^{L}$	-0.024 (-0.038)	0.003 (-0.006)	0.133 (0.057)	0.04 (0.019)	0.345 (0.344)	0.211 (0.21)	0.94	0.96
${\hat{\theta }}^{P}$	-0.016 (-0.016)	0.012 (0.003)	0.148 (0.059)	0.045 (0.02)	0.384 (0.382)	0.222 (0.222)	0.96	0.97
${\hat{\theta }}^{MWE}$	-0.024 (-0.035)	0.007 (-0.003)	0.134 (0.058)	0.04 (0.019)	0.341 (0.341)	0.207 (0.207)	0.94	0.96
${\hat{\theta }}^{JWE}$	-0.025 (-0.035)	0.007 (-0.002)	0.135 (0.058)	0.039 (0.018)	0.341 (0.341)	0.206 (0.207)	0.94	0.96

^1^ ${\hat{\theta }}^{L}$: the estimator by [13]; ${\hat{\theta }}^{P}$: the estimator by [17]; ${\hat{\theta }}^{MWE}$: the weighted estimator by the marginal approach; ${\hat{\theta }}^{JWE}$: the weighted estimator by the joint approach

^2^ median of difference of the estimator from true value

^3^ mean squared error

^4^ median of squared error

^5^ mean of standard error

^6^ median of standard error

^7^ 95% coverage rate

In these simulation results, we can see that our weighted estimators have good results. In both cases, bias and mean squared error of our new estimator has similar performance compared to the estimators by [13] and [17]. Mean of standard errors and median of standard errors are smaller than the estimators by [13] and [17]. Moreover, computation results for the median of difference of the estimators from true value and the median of squared error imply that our proposed estimator is comparable with the estimators from the original methods.

In the first simulation setting, the difference of standard error between our proposed estimator and ${\hat{\theta }}^{L}$ is bigger than the one between ${\hat{\theta }}^{P}$ and the proposed estimator. In the second simulation setting, the phenomenon is the opposite. Furthermore, in the first simulation setting, ${\hat{\theta }}^{P}$ has lower standard error on average than one of ${\hat{\theta }}^{L}$ while ${\hat{\theta }}^{L}$ have better efficiency (with respect to standard error) than ones by ${\hat{\theta }}^{P}$ in the second simulation setting. This simulation result verifies our claim, which means that neither estimator is better than another. Our proposed estimator takes advantage of smaller standard error with achieving small bias and correct coverage except *N* = 150 with *σ* ^2^ = 1 in the second simulation setting. In this scenario, empirical coverage of proposed estimators is lower than nominal 95% coverage. This is due to low coverage of ${\hat{\theta }}^{L}$. Since we combine ${\hat{\theta }}^{L}$ and ${\hat{\theta }}^{P}$, if one of them has low coverage, it is highly likely that the coverage of weighted estimator may also be below the nominal coverage.

## Real data analysis

We applied our method to data from the AIDS Clinical Trial Group (ACTG) Study 364 [1], which was used in [17]. This multicenter randomized study investigated patients whose plasma RNA level is at least 500 copies per ml. Subjects were assigned to three treatments, nelfinavir (NFV), efavirenz (EFV), and combination of nelfinavir and efavirenz (NFV + EFV). Details about this study can be found in [1].

The two failure times are time to HIV RNA level greater than 2000 copies per ml and time to withdrawal of study. Let *X* be the first time when HIV RNA level is greater than 2000 copies per ml and *D* be time to withdrawal of study. We considered four covariates and 194 observations. *Z*_1_ takes value 1 if a patient receives EFV and 0 otherwise. *Z*_2_ takes value 1 if a patient receives NFV + EFV and 0 otherwise. *Z*_3_ is New3TC, which takes value 1 if lamivudine is given as a new nucleoside analogue therapy to a patient and 0 otherwise. *Z*_4_ is logarithm of RNA level at the start of the study.

Table 5 and Table 6 show the point estimates and standard errors of $\hat{\eta }$, ${\hat{\theta }}^{L}$, ${\hat{\theta }}^{P}$, ${\hat{\theta }}^{MWE}$ and ${\hat{\theta }}^{JWE}$. Our method works well for the models with and without New3TC on all covariates. Some variables are seen to be statistically significant based on the weighted estimator while they are not by [13] or [17]. For example, let’s consider effect of EFV to the time to first virologic failure. By Table 6, the estimated effect by using approach of [13] is 0.475 and its standard error is 0.250. From approach of [17], an estimate is 0.464 and its standard error is 0.281. Based on the fact that estimators are asymptotic normal, from Wald test using [13] and [17], EFV is not a statistically significant variable on 5% significant level. On the other hand, a weighted estimate using first approach is 0.471 and its standard error is 0.222. In this case, EFV is a statistically significant variable on 5% significant level.

**Table 5:** Point estimates with standard errors of covariates in AIDS study for model without New3TC (Standard errors are shown in parenthesis).

Covariates	$\hat{\eta }$	${\hat{\theta }}^{L}$	${\hat{\theta }}^{P}$	${\hat{\theta }}^{MWE}$	${\hat{\theta }}^{JWE}$
EFV^1^	0.753 (0.339)	0.115 (0.219)	0.375 (0.269)	0.168 (0.206)	0.2 (0.205)
NFV^2^+ EFV	0.674 (0.255)	1.128 (0.239)	1.091 (0.309)	1.120 (0.222)	1.114 (0.222)
log(RNA)^3^	-0.544 (0.154)	-0.464 (0.215)	-0.531 (0.169)	-0.507 (0.163)	-0.511 (0.162)

^1^ efavirenz

^2^ nelfinavir

^3^ logarithm of RNA at the start of the study

**Table 6:** Point estimates with standard errors of covariates in AIDS study for model with New3TC (Standard errors are shown in parenthesis).

Covariates	$\hat{\eta }$	${\hat{\theta }}^{L}$	${\hat{\theta }}^{P}$	${\hat{\theta }}^{MWE}$	${\hat{\theta }}^{JWE}$
EFV^1^	0.770 (0.278)	0.475 (0.250)	0.464 (0.281)	0.471 (0.222)	0.471 (0.222)
NFV^2^+ EFV	0.650 (0.260)	1.353 (0.277)	1.246 (0.338)	1.333 (0.263)	1.317 (0.261)
New3TC^3^	0.927 (0.355)	1.449 (0.296)	1.374 (0.328)	1.431 (0.267)	1.420 (0.261)
log(RNA)^4^	-0.631 (0.183)	-0.654 (0.289)	-0.661 (0.218)	-0.659 (0.216)	-0.660 (0.215)

^1^ efavirenz

^2^ nelfinavir

^3^ lamivudine as new nucleoside analogue therapy

^4^ logarithm of RNA at the start of the study

**Fig 1:**
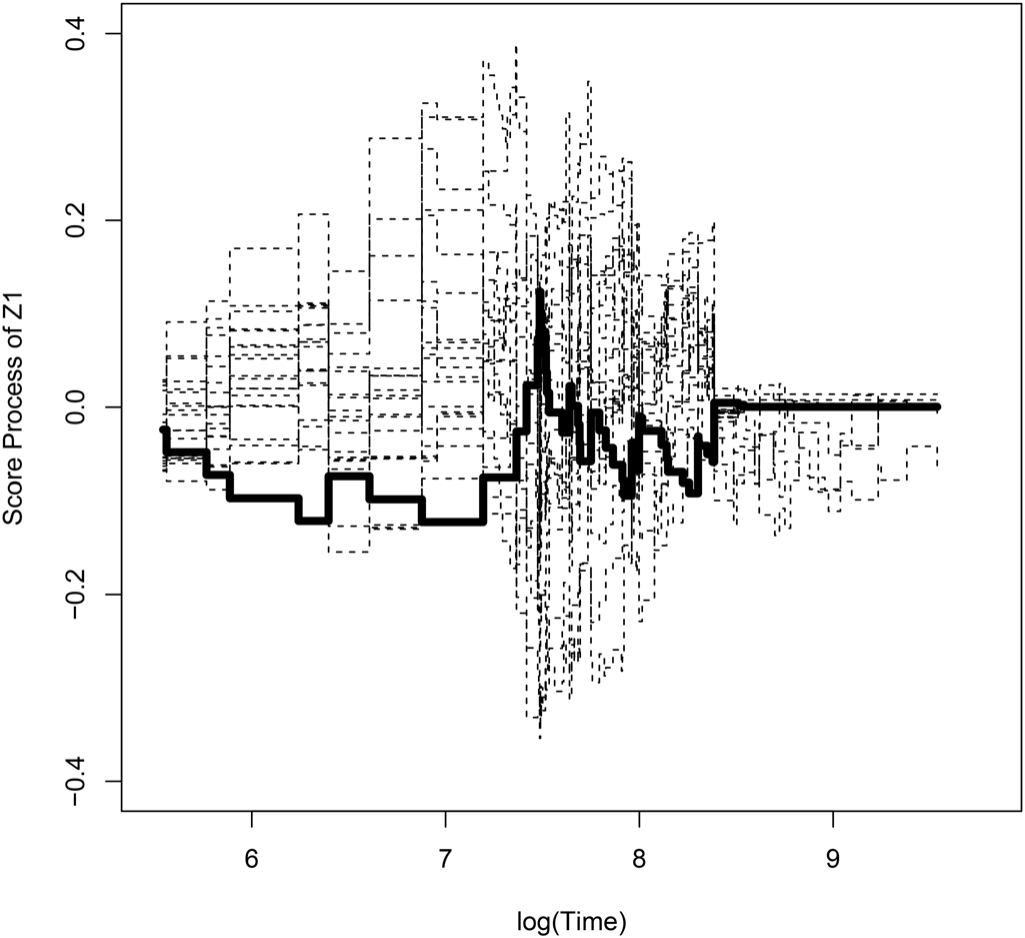
Plot of observed score process and bootstrapped processes of time to withdrawal of study with respect to *Z*_1_. **The thickline is observed process and the dashed lines are bootstrapped processes**.

**Fig 2:**
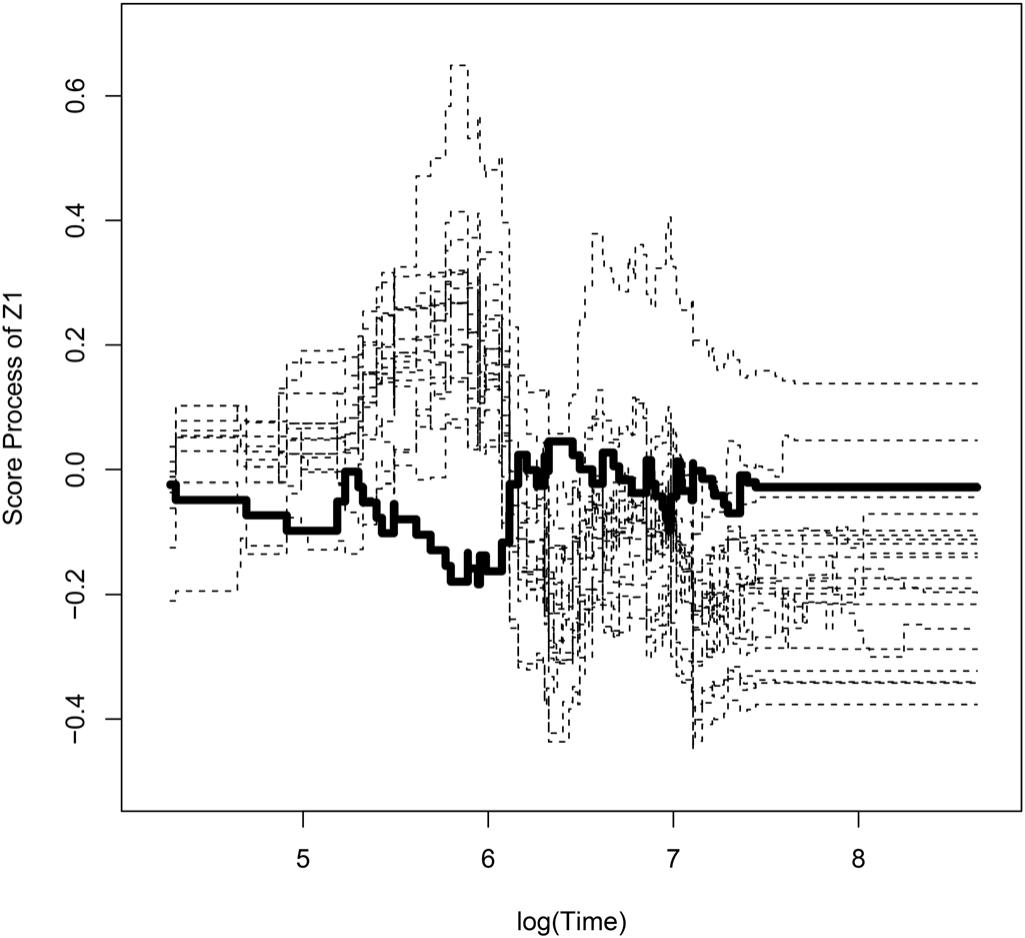
Plot of observed score process and bootstrapped processes of time to first virologic failure using ${\hat{\alpha }}^{L}$ with respect to *Z*_1_. **The thickline is observed process and the dashed lines are bootstrapped processes**.

**Fig 3:**
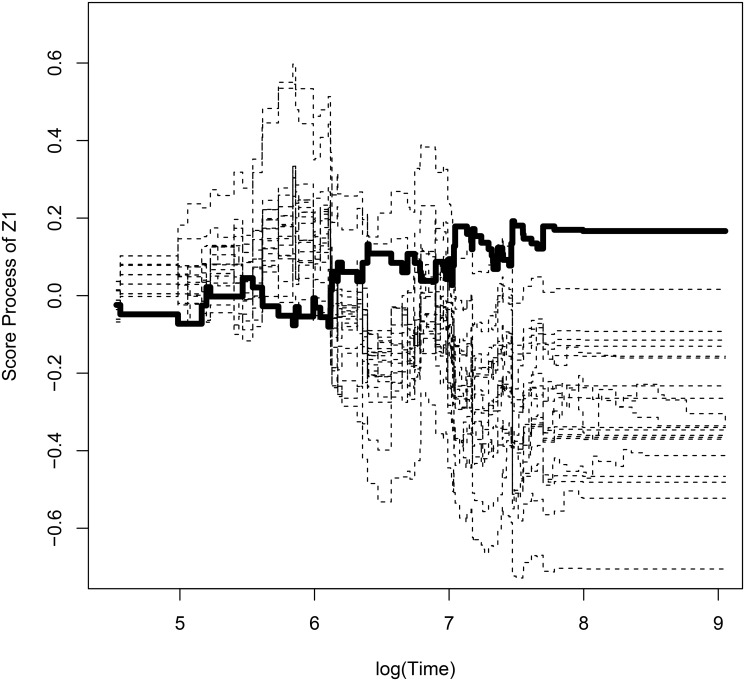
Plot of observed score process and bootstrapped processes of time to first virologic failure using ${\hat{\alpha }}^{P}$ with respect to *Z*_1_. **The thickline is observed process and the dashed lines are bootstrapped processes**.

Observed score process with bootstrapped processes for withdrawal of study with respect to *Z*_1_ is shown in Fig 1. Fig 2 and Fig 3 show observed score processes and bootstrapped processes of the first virologic failure using ${\hat{\alpha }}^{L},{\hat{\alpha }}^{P}$ with respect to *Z*_1_. These three plots are based on the model without New3TC. They are fluctuating around zero, so it seems that there is no graphical evidence for lack of fit. The p-value for the lack of fit tests of withdrawal is 0.952 and the first virologic failure using ${\hat{\alpha }}^{L}$ and ${\hat{\alpha }}^{P}$ are 0.918 and 0.959 respectively. With graphical checking, p-value indicates that there is no evidence for violation of the model assumption.

For purposes of interpretation, since *D* represents a standard survival time, the interpretation of $\hat{\eta }$ is in terms of covariate effect for survival time. However, since the observed time for *X* depends on *D*, interpretation of $\hat{\theta }$ is difficult. One way to interpret $\hat{\theta }$ is to assume that *D* does not exist and interpret the effect of $\hat{\theta }$ on *X* only. This approach is possible if there exists a reasonable extrapolation mechanism for *X* [18]. However, considering the estimation structure for ***θ***, it is difficult to separate effect of $\hat{\theta }$ to *X* from effect of $\hat{\eta }$ to *D*.

## Discussion

In this paper, we have proposed optimal estimators using combinations of the two estimators from [13] and [17]. Our methodology can be extended to a case of recurrent event with dependent censoring, which is extensively studied [6], [7], [10]. We are currently working on this extension.

Optimality of the estimator has been discussed in other contexts. Recently, there is a publication that proposed optimal additive functions based on score functions [14]. The main point of their method is to combine unbiased estimating functions. In our case, this would be combining estimating equations and new solution can be obtained by this estimating equation. Comparing performance of this solution and our proposed estimator is of interest. This will be left open to future research.

Another way of achieving optimality is to use generalized method of moment estimator [8]. This estimator is a linear combination of estimating functions [19]. In this case, the estimating functions have a greater dimension than the dimension of the parameter vector. The optimality is achieved by the linear combination. It is shown that the estimator from this linear combination of estimating functions is consistent and asymptotically normal [8]. In the literature of statistics, this idea is applied to generalized estimating equations [19]. The estimating functions proposed by [19] are called quadratic inference function. Recently, the quadratic inference function is applied to Cox model [26].

[8] and [19] derived new estimating functions, while we combined two estimators directly. This idea of the generalized method of moments is very appealing, but the estimating functions of [13] and [17] are nonsmooth. Finding derivative for the linear combination of the estimating functions, which is a key in generalized method moments, is challenging for our work because we cannot find the derivatives in the estimating functions proposed by [13] and [17]. Applying the idea of [8] to AFT model will be interesting future research.

Our estimating equations to obtain estimators involve nonsmooth functions of ***η*** and ***α***. Many literatures used a linear programming approach for estimating ***θ*** [3], [11]. However, this linear programming method is very slow for computing estimators of ***θ***. Thus this approach is very inefficient when implementing to solve (Eq 11) for estimation of **Σ**. Recently, an approach called a derivative free-spectral algorithm for nonlinear equations (DF-SANE) was proposed [12], and there is a publication that showed that this algorithm is better than the linear programming method using an example of estimating parameters of AFT models under independent censoring. [21]. However, under dependent censoring, the artificial censoring term leads to numerical instability in estimating parameters and calculating resampled estimators. Moreover, this algorithm does not converge well under default tolerance settings using DF-SANE [21]. Thus using this algorithm requires changing the tolerance level. Developing efficient numerical algorithms for estimating parameters is an important topic for future research.
